# Multiple remote epidural hematomas following pineal gland tumor resection

**DOI:** 10.4103/1817-1745.66674

**Published:** 2010

**Authors:** Jeong-Wook Lim, Seung-Hwan Yang, Jong-Soo Lee, Shi-Hun Song

**Affiliations:** Department of Neurosurgery, Daejeon Sun General Hospital, Daejeon, Korea; 1 Department of Neurosurgery, ChungNam National University Hospital, Daejeon, Korea

**Keywords:** Hydrocephalus, multiple epidural hematoma, pineal tumor

## Abstract

In cases of pineal tumor combined with obstructive hydrocephalus, preoperative ventriculostomy or ventriculoperitoneal shunting is typically required prior to tumor resection. The objectives of preoperative ventriculostomy are gradual reduction of intracranial pressure and consequent preoperative brain protection. Here we report a case of pineal tumor resection with preoperative ventriculostomy that was complicated by multiple epidural hematomas. While postoperative intracranial hemorrhage may occur at any site, it is rare in those areas remote from the operative field. In the present case, multiple remote sequential epidural hematomas developed following resection of a pineal gland tumor. We also discuss the pathophysiologic mechanisms and provide a literature review.

## Introduction

Postoperative intracranial hemorrhage is a serious complication of intracranial procedures. Hyatai reported that the incidence of postoperative hematoma formation following brain surgery was 2.1%.[1] Of these, 64% were intracerebral, 18% subdural, 14% epidural, and 4% cerebellar.[1] Postoperative intracranial hemorrhage mostly occurs within the operative site. Remote, sequential, multiple epidural hematomas (EDHs) following shunting has only been reported once before.[2] Here we report the formation of multiple EDHs remote from the site of craniotomy following pineal tumor resection. The patient underwent successful reoperation for EDH at four different sites. We discuss the mechanism of development and suggest techniques for avoidance of such complications.

## Case Report

A 9-year-old boy presented with progressive gait disturbance and right-sided weakness of 1 year’s duration. His past medical history was significant for hydrocephaly, and he had experienced several episodes of tonic-type seizures prior to the age of 3 years. He had demonstrated clinical improvement without specific antiepileptic therapy.

Upon admission, he was diagnosed with a pineal gland tumor on the basis of the physical examination and radiologic findings [Figure 1]. The tumor was resected by the supracerebellar infratentorial approach, with the patient in the Concord position. A preoperative extraventricular drainage to the right side of Kocher’s point was performed and cerebrospinal fluid (CSF) was noted to drain slowly. The dura was opened, the cisterna magna was dissected, the bridging veins draining to the transverse sinus were removed, and the cerebellum was allowed to move inferiorly. The mass was then completely resected without complication. Pathology confirmed the presence of a mature teratoma.

**Figure 1A:**
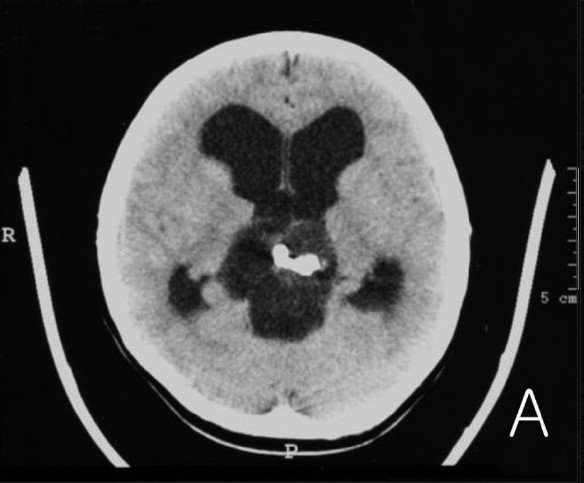
Preoperative brain computed tomography (CT) image showing a calcified cystic mass in the pineal gland (A)

**Figure 1B:**
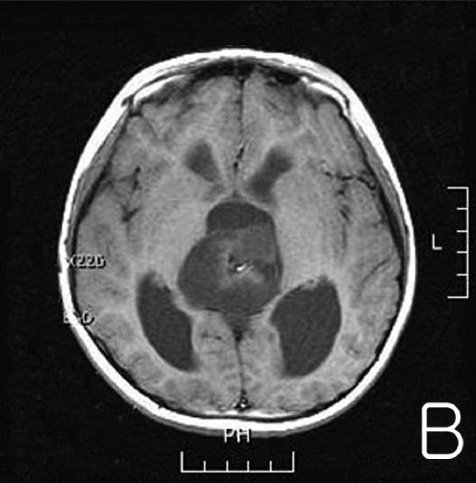
T1-weighted gadolinium-enhanced axial magnetic resonance (MR) images (B) showing an inhomogenous enhancing mass in the pineal gland

**Figure 2:**
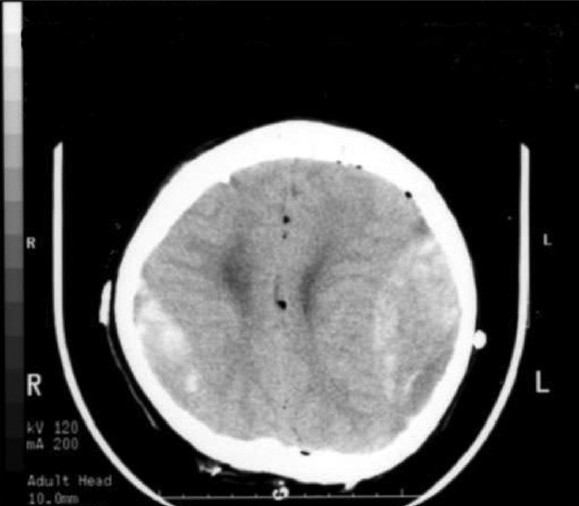
Immediate postoperative brain CT showing acute EDHs in bilateral frontoparietal regions

**Figure 3:**
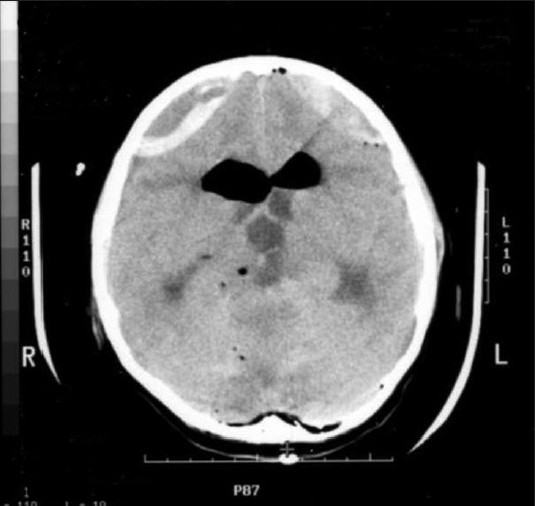
Brain CT showing acute EDHs following reoperation in the bifrontal areas and pneumoventricle in bilateral frontal horns

**Figure 4:**
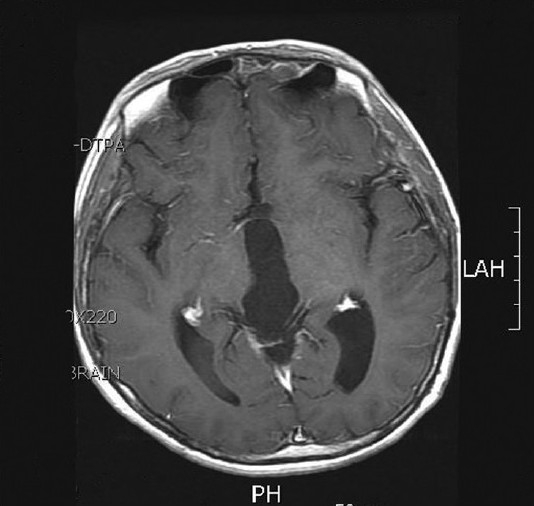
Magnetic resonance (MR) image 3 years following tumor resection showing no evidence of recurrence

The patient was noted to be lethargic postoperatively and, when a subsequent brain CT scan showed bilateral parietal EDHs, emergency craniotomy was performed for their removal [Figure 2]. Follow-up brain CT after reoperation demonstrated the presence of small bifrontal EDHs [Figure 3]. The mentality was recovered but the patient complained of continuously headache and gait disturbance and, therefore bilateral frontal twist trephination and hematoma removal was performed 2 weeks later. The headache, gait disturbance, and right hemiparesis improved following this and there has been no evidence of tumor recurrence during the 3-year follow-up period [Figure 4].

## Discussion

Postoperative intracranial hematoma is one of the most serious complications of intracranial operations. Palmar *et al*. reported that the most common procedure leading to the formation of postoperative hematoma was meningioma resection, with a rate of 6.2%; this was followed by craniotomy for trauma (at 3.7%), aneurysm surgery (at 2.6%), and intrinsic supratentorial tumors (at 2.2%).[3] Postoperative hematomas were noted to be intraparenchymal in 43% of cases, subdural in 5%, extradural in 33%, mixed in 8%, and confined to the superficial wound in 11%.[3] The rate of postoperative EDHs has been reported to be approximately 1% at the site of craniotomy; however, multiple remote EDHs are quite rare.[4] Postoperative EDHs may produce serious complications, given that the neurologic symptoms may be seemingly unrelated to the original operative site. Postoperative remote EDHs following ventriculoperitoneal shunting and cranial operation have been reported earlier in the literature.[5–10]

The pathophysiology of remote intracranial hemorrhage following craniotomy suggests that sudden decreased intracranial pressure, unequal distribution of intracranial pressure between compartments with a consequent shifting of brain parenchyma, massive drainage of CSF, or a bleeding tendency may all serve as potential triggers.[5,7,8,10,11] In cases of remote EDH following ventriculoperitoneal shunting, the massive drainage of CSF is an important causative factor.[2,8] Unclear sentence was changed to : Young age is another contributory factor, the adhesion between the dura and the cranium could become weak with age. A prone position and severe adhesion between the arachnoid and pia mater are also important pathophysiologic factors.[2,8] Yacubian *et al*. reported that remote EDH is influenced by multiple factors, including brain shifting, dural detachment due to improvement of blood flow in the contralateral compressed dural vessel, and massive drainage of CSF.[10] Many pineal gland masses are associated with hydrocephalus secondary to compression of the aqueduct of Sylvius. If the pineal gland mass results in a noncommunicating hydrocephalus early in life, craniocephalic disproportion ensues. The ratio of brain parenchyma to intracranial volume is relatively small in this form of hydrocephalus, and the greater the craniocephalic disproportion the larger the potential EDH. Park reported that distant EDH following ventriculoperitoneal shunting commonly occurs in the frontal area due to the weak adhesion between the dura and calvarium in this location.[2]

There are many potential contributing factors for the formation of remote, multiple, EDHs in the present case. First, massive removal of CSF by preoperative extraventricular drainage and opening of the cistern during the arachnoid dissection resulted in a symmetric decrease in intracranial pressure. Second, removal of the centrally located mass with the patient in the prone position caused brain shifting. Finally, the weak attachment between the dura and the cranium, as well as the high compliance of the dura, resulted in easy detachment.

Bae *et al*.[5] suggested that sudden perioperative lowering of intracranial pressure by massive CSF drainage should be avoided and that the head should be lowered when the patient is in the prone position during the operation to avoid such complications.[5] Preoperative ventriculoperitoneal shunting should then be performed 1 or 2 weeks prior to tumor resection. Brain CT is mandatory in the presence of postoperative lethargy, a new neurologic deficit, prolonged decreased mentation, or neurologic deficits unrelated to the operative field.

Conservative management may be the choice of treatment in cases of small EDH or mild symptoms; however, large EDHs, as were present in this case, require urgent surgical intervention. As rapid hematoma removal may induce contralateral hemorrhage, subsequent brain CT should be performed.

## Conclusion

Here, we report a case of pineal tumor combined with hydrocephalus, complicated by multiple postoperative EDHs. The mature teratoma of the pineal gland is considered curable by surgical resection. In cases combined with severe hydrocephalus, preoperative ventriculostomy may be performed. A gradual reduction of intracranial pressure may prevent sudden ventricular collapse. Postoperative intracranial hemorrhage must be evacuated immediately in symptomatic patients.
